# Understanding Health-Related Motivations for Urban Food Self-Production in the Light of Semantic Fields Analysis

**DOI:** 10.3390/nu16101533

**Published:** 2024-05-20

**Authors:** Ewa Duda

**Affiliations:** Institute of Education, Maria Grzegorzewska University, 02-353 Warsaw, Poland; eduda@aps.edu.pl

**Keywords:** environmental literacy, food-growing intention, green transformation, health education, healthy foods, public health and nutrition, pro-environmental behaviors, technology-enhanced education, urban gardening, Urban Living Lab

## Abstract

One of the contemporary challenges facing urban areas is the necessity to identify novel approaches to resident involvement in solution creation, with a particular focus on ensuring the best possible nutrition. By investigating the process of co-participation of city dwellers in a unique education project, this paper aims to gain a deeper understanding of the health-related motivations that underpin the decision of early adopters of the implemented technological innovations to join the social experiment. The qualitative study employed purposive sampling and in-depth interviews conducted in two waves, the first between October and November 2022 and the second between September 2023 and January 2024. The study comprised 42 participants drawn from two communities of residents in Łódź and Warsaw, Poland. Transcriptions of the interviews were carried out using semantic field analysis, employing a quantitative approach that counts the frequency of keyword occurrences. Three categories of semantic fields were identified: associations, oppositions, and actions toward the subject, including positive, neutral, and negative temperatures. The findings demonstrate that the health concerns of residents are a pivotal factor in their decision to participate in urban food self-production initiatives, given their limited access to nutritious and healthy vegetables. This is related to several factors, including restrictions related to urbanization and the displacement of local suppliers, lifestyle, and the fast pace of urban life. The dissemination of innovative solutions for growing food in urban environments could, therefore, facilitate awareness-raising and motivation to alter the dietary habits of inhabitants.

## 1. Introduction

The topic of urban development has recently gained renewed attention, particularly in the context of creating green, competitive, and inclusive living spaces, as well as providing quality food [1]. That is particularly relevant given the increasing prevalence of actions that contradict the concept, such as the removal of green spaces to facilitate the construction of additional buildings or the creation of areas that, due to the predominance of concrete, appear to be more easily maintained. The increasingly common elimination of greenery from urban spaces, including fruit orchards and vegetable gardens, not only results in limited access to nature but also has wider implications for the environment. There is a lack of such green spaces that could effectively promote a positive attitude towards gardening and serve as venues for organizing educational interventions promoting food security, diverse and balanced nutrition, and health, while simultaneously maximizing the health benefits associated with plant cultivation in urban spaces [2].

This study forms part of a larger project that is investigating novel approaches to engaging urban residents in the development of solutions that optimize their access to nutritious foods while addressing the challenges posed by climate change. The intention of the paper is to contribute to the ongoing academic discussion on fostering green urban development by elucidating the health-related motivations that underpin the decision of early adopters of the implemented technological innovations to join the social experiment. The qualitative research conducted among the participants of the presented project aimed to ascertain how they determine health issues, their nutrient supply needs, and their motivations, attitudes, intentions and expectations towards the educational intervention tools created. The findings of the study can inform the design and development of innovative educational tools that promote pro-environmental behavior and foster the development of environmental literacy [3]. This can bring multiple benefits to society and contribute to the creation of more sustainable future cities.

This article comprehensively analyzes the statements of participants in a unique educational experiment, drawing insights from interdisciplinary research, the results of which are presented in subsequent sections of the article. The “Current Study” section introduces theoretical aspects related to the article’s topic, introducing the reader to the subject of urban gardening, its connections to health issues, food security, and the introduction of innovative educational tools for the development of urban gardening. The “Materials and Methods” section defines the overarching goals and theoretical frameworks underlying this study, outlining its assumptions and course. The “Results” section delves into a meticulous analysis of the participants’ statements, emphasizing health issues that emerged during responses to questions about participants’ previous gardening experiences and their expectations for upcoming activities. The following section elaborates on the research findings in relation to the achievements of other authors and presents practical implications and recommendations for the project implementation team, as well as other researchers and practitioners interested in the research topic. The final section briefly summarizes the study, delineates its main contribution to the development of the discipline, presents the study’s limitations, and outlines plans for further research.

## 2. Current Study

Urban gardening, often associated with aesthetics and relaxation [4,5], has become a significant aspect of public health with rapid urbanization and population growth [6]. The development of urban gardening can significantly contribute to improving the quality of life for residents, supporting efforts to enhance societal well-being, including health [7]. Improving air quality, promoting physical activity, and ensuring access to healthy food are key aspects that can be incorporated into the development of urban green spaces.

Urban gardening promotes physical activity and a healthy lifestyle [8]. Cultivating vegetables, fruits, and herbs in urban allotment gardens or on balconies encourages greater activity, including outdoor activities. Regular gardening can improve physical fitness and stamina, and reduce stress, contributing to overall physical and mental health improvement [9]. Community urban gardens, squares, or parks with edible gardens provide residents with access to fresh and healthy agricultural products [10]. Consuming fresh fruits and vegetables from one’s cultivation can contribute to maintaining a balanced diet rich in vitamins, minerals, and antioxidants, thereby improving overall health and immunity [11].

However, despite these benefits, there are also health challenges associated with urban gardening. One of the main issues is soil contamination. Cities often have chemical pollutants, such as heavy metals and pesticides, which can accumulate in the soil and be transferred to cultivated plants. Consuming such products can pose a health risk to people [12]. Another challenge is the presence of pests and plant diseases. In urban conditions, where gardens are usually densely packed, there is a greater risk of problems related to pests and plant diseases. The use of pesticides to control these pests can have negative effects on human health and the environment [13]. There is also a risk associated with the quality of water used for watering plants. In some urban areas, water quality may be low due to chemical and biological pollutants. Using such water for watering plants can lead to the contamination of agricultural products and pose a health risk [14].

In response to these challenges, efforts are being made to introduce innovative solutions that would mitigate the negative impact of urban gardening on human health. One of them is hydroponic food cultivation [15]. One of the major advantages of hydroponics is its efficiency in resource utilization. This may be a significant factor in encouraging urban dwellers to utilize hydroponic techniques for the cultivation of food. In traditional agriculture, a large amount of water is lost through evaporation and absorption by the soil, whereas in hydroponics, water is directly supplied to the plant roots. This process allows for water conservation, which is essential not only in the context of human health but also in the context of climate change and global water resource shortages [16].

Secondly, the pursuit of food self-sufficiency can serve as a motivating factor for the utilization of hydroponic cultivation [17]. In urban areas where access to fresh produce may be limited, the ability to cultivate vegetables or herbs in one’s own home or on a balcony can be a highly appealing proposition. The implementation of hydroponics allows for the efficient management of space and resources, which is of particular importance in urban environments where land availability is limited. Future users may be encouraged by the fact that plants in hydroponics can be grown in a variety of locations, including indoors, on rooftops, in vacant lots, or even in desert areas where soil conditions are unfavorable. This gives the opportunity to produce food locally, reducing the need for long-distance transportation and the associated greenhouse gas emissions.

An additional rationale for utilizing alternative methods of cultivating food in urban environments is the ability to exert control over the environmental conditions that influence plant growth. Technological advances, including the development of advanced hydroponic systems, also influence the motivation to use this form of cultivation. The advent of modern technology has enabled the automation of the agricultural growing process, rendering it more accessible and easier to manage even for individuals lacking prior farming experience. In hydroponics, pH level, nutrient solution composition, temperature, and humidity can be precisely controlled, allowing for optimal growth conditions for different plant species. This, in turn, leads to faster growth and better crop quality. The products in question are typically greener, free of harmful chemicals and pesticides, and therefore safer for human health, more palatable, and nutritionally superior [18].

However, hydroponics is not without drawbacks, negatively influencing the decision to use it. One of the main issues with this system is the initial investment cost. Building and maintaining a hydroponic system can be more expensive than traditional soil cultivation, especially initially. The hydroponic installation constituted a research contribution, funded by a grant, which enabled the realization of this unique project. Consequently, an analysis of the cost-effectiveness of hydroponic solutions in the city was not carried out as part of the project. However, it is worth noting that these issues are important when implementing innovative solutions requiring a significant financial contribution. Another challenge is maintaining balance in the nutrient solution. The regular monitoring and adjustment of pH level and nutrient solution composition are necessary to provide plants with adequate nutrition. Improper balancing can lead to nutrient deficiencies or excesses, which in turn can negatively affect plant growth [19].

Despite the aforementioned limitations, the implementation of urban farming through hydroponic installations can provide a response to the challenges faced by contemporary cities in ensuring healthy and sustainable food for increasingly diverse and growing urban populations. Urban hydroponic farming represents an innovative strategy that not only promotes local food production but also alters the way residents make decisions regarding their diet and healthy lifestyle. It is therefore important to consider whether residents are inclined to adopt this strategy.

One of the key motivational factors could be the growing health awareness among urban residents. There is a growing recognition that a healthy diet based on fresh, local products can contribute to overall health improvements, reduce chronic disease risks such as obesity, diabetes and heart diseases, or support preventive actions. Previous studies on traditional farming have demonstrated that urban food cultivation in community gardens, school gardens, and backyard gardens provides residents with direct access to fresh vegetables, fruits, and herbs, which can encourage them to make healthier dietary choices [20].

The pursuit of food self-sufficiency can be a significant motivating factor for engaging in traditional urban food cultivation. The capacity to cultivate one’s own vegetables or fruits engenders a sense of autonomy and gratification. Individuals value the ability to control the quality of their food and to avoid the use of artificial pesticides or herbicides, which are often employed in commercial agriculture. Fresh, locally grown products are richer in nutrients and vitamins than those available in supermarkets, which are often subjected to lengthy storage and transportation processes. Consuming fresh fruits and vegetables from one’s own garden is beneficial to one’s health, which in turn affects the overall physical and mental well-being of residents [21].

In the case of traditional gardens, urban food cultivation can become a means of spending active time outdoors for a significant proportion of the population. The act of working in a garden provides a multitude of benefits, including physical exercise, relaxation, and a means of disconnecting from the urban environment. The combination of physical activity and food production serves as a motivating factor for individuals to engage in regular care of their garden or plot, as well as their health [22].

Building a local community is also identified as a motivation that attracts individuals to engage in food production within urban areas. The health benefits associated with the consumption of collectively cultivated products contribute to the overall improvement of residents’ health and facilitate the modification of their dietary habits. Urban food cultivation is therefore not only a means of attaining a healthier lifestyle but also fosters the development of a health-oriented local community. Those who engage in this activity frequently establish connections with their neighbors and other enthusiasts of a healthy lifestyle. Community gatherings in community gardens or local farmers’ markets facilitate social integration and the exchange of experiences [23].

The main goal of the presented study is therefore to gain a deeper understanding of the health-related motivations that influenced the decision of early adopters of technological innovations oriented towards self-food production. The research will aim to examine how they perceive health issues and their nutritional needs, and through them motivations, attitudes, intentions, and expectations related to the educational intervention tools being created. The intention of the study is therefore to deepen the understanding of whether the health-related motivations underpinning the unique project are analogous to those of conventional urban food gardens, or whether motivations and attitudes diverge when urban food growing is linked to innovative technology. These objectives are expressed through the following research questions:

RQ1—How do positive-emotional networks of semantic fields reveal the perception of health-related motivations by city residents planning to engage in innovative food self-production?

RQ2—How do neutral-emotional networks of semantic fields reveal the perception of health-related motivations by city residents planning to engage in innovative food self-production?

RQ3—How do negative-emotional networks of semantic fields reveal the perception of health-related motivations by city residents planning to engage in innovative food self-production?

## 3. Materials and Methods

### 3.1. Theoretical Framework of the Study

The study was conducted based on Rogers’ Diffusion of Innovations Theory [24]. This approach focuses on the experiences of early adopters, who are crucial in the diffusion of innovations. It is through their openness that new, innovative ideas are tested and then transferred between different peer groups. Early adopters are a social group that engages in the acceptance of new ideas or technologies, often preceding the majority of society. They stand out for their readiness to test and their openness to innovations. They frequently act as opinion leaders within their communities and derive satisfaction from experimenting with new solutions. An important factor determining the behavior of early adopters is their ability to assess the value of innovations. Perceived value, or the subjective belief in the utility of innovations, plays a crucial role in the adoption process. Early adopters are capable of quickly evaluating the benefits of a new technology or idea and making swift decisions regarding its adoption. Their willingness to try something new often stems from a belief in the potential benefits of a given solution [24].

Furthermore, social networks play a significant role in shaping the behaviors of early adopters. These individuals often connect with others sharing common goals, facilitating information exchange, and encouraging decisions to adopt new innovations. Early adopters can also be inspired by other members of their social group who are more open to novelties and serve as role models. However, early adopters bear greater risks than those who wait for later stages of innovation diffusion. New technologies or ideas may still be underdeveloped or unreliable, carrying a risk of failure. Additionally, the early adoption of innovations can be time-consuming and may require additional effort in learning and adapting to new practices. Overcoming these initial challenges, the engagement of early adopters can accelerate societal acceptance of innovation, creating a snowball effect where positive experiences attract subsequent groups to adopt the innovation. Simultaneously, their actions can provide valuable feedback to innovation creators, enabling the improvement and customization of products to meet user needs [24].

### 3.2. Procedure, Participants

Addressing the challenges of contemporary cities, this study is part of a project aimed at implementing innovative tools based on sustainable food consumption and production patterns, promoting healthy dietary habits, social inclusion, and reducing food waste. The project is likely the first such experiment conducted in Poland. As part of a controlled social experiment carried out in a selected residential block, residents participate in hydroponic food cultivation located in the corridors of their block [25]. Due to the high installation costs, the project involves 20 residents responsible for individual hydroponic cabins.

The study involved two groups of respondents. The first group of residents selected for the project originated from Łódź, but due to legal constraints pertaining to the guarantee of an inhabited building, the experimental phase in this location was not feasible. Consequently, the first research group did not participate in the experiment, and a second residential community had to be selected. The second group of residents came from the city of Warsaw. Both cities are among the largest cities in Poland and are located in central Poland. The experimental phase of the project took place in a selected residential block in Warsaw, in the Mokotów district.

The study participants are owners or tenants of residential premises who expressed a willingness to participate in an innovative social experiment. Due to the need to understand their needs and expectations regarding project activities, the research sample was purposively selected. Here, 37 individual in-depth interviews lasting from 1.5 to 2 h were conducted with them. The interviews were conducted by a team of four professionals, all members of the research team. Two of the team members hold doctoral degrees, while the remaining two are PhD candidates. Prior to their participation in the interviews, the participants were informed of the nature of the study and gave informed consent to participate in the interviews. Participants were compensated with a monthly amount of approximately EUR 22 for participating in project activities. The first set of interviews was conducted from October to November 2022, while the second set was conducted from September 2023 to January 2024. The time gap between interviews was due to the need to select a second project site that met certain technical requirements.

The interviews were conducted prior to the commencement of the experimental phase of the project. At this stage, the participants had limited knowledge of the implementation of the project. They were aware of the purpose of the experiment and the benefits they would receive from participating in it. The project required the insertion of 20 hydroponic cabins in the corridors of the block, the installation of a photovoltaic system on the roof of the building to power the cabins and the installation of a water supply system for the cabins with the possibility of using rainwater. At the finished stage of the project, the residents’ community was presented with a choice: to keep the hydroponic cabins or to uninstall them. In contrast, the photovoltaic installation was to be permanently installed in the block. The planned interviews were intended to facilitate an early understanding of the project applicants, regarding their expectations, intentions, and needs, prior to the commencement of the experimental activities.

The interviews covered the following thematic areas: (1) introductory information, (2) neighborly relations, (3) issues related to participation in the project, (4) food waste. A total of 42 respondents participated in the interviews (*M*age = 43.7, *SD* = 14.6, range 27–78), including 26 women (*M*age = 46.1, *SD* = 16.1, range 27–78) and 16 men (*M*age = 40.1, *SD* = 11.8, range 28–77). Eighteen participants were childless, eighteen had one child, including twelve minors, four participants had two children, including three minors, and one participant had three minors. Four participants had completed secondary education, 37 participants had obtained university degrees, and one participant did not specify their level of education. Four participants had received education in agriculture, while six participants had received education in healthcare. The remaining participants represented a range of other professions. Of the 19 individuals who participated in the initial interview phase and were subsequently deemed eligible for inclusion in the project, 19 ultimately did not take part in the project. The remaining 23 participants from the second wave of interviews proceeded with the project. The participants constituted the full experimental group.

### 3.3. Data Analysis

Participants’ statements were analyzed and interpreted according to the semantic field analysis method [26]. This method involves selecting the object of study represented by a keyword (in this study, the keyword is “health”), and then analyzing statements containing this word (the so-called semantic field) based on their belonging to the selected network of meanings. This method allows for a more in-depth analysis of respondents’ perceptions of the issues relevant to the study. While this method is not commonly employed in non-linguistic disciplines, a number of valuable analyses conducted using it can be found in the literature [27]. The presented study represents a novel research approach, employing methods originally developed for other disciplines in new areas.

Robin [26] identifies six distinct networks of meanings. Three of them were selected for analysis in the context of this study: (1) network of associations with the analyzed keyword “health”; (2) network of opposites to the keyword “health”; (3) network of actions towards the subject, i.e., a set of words or phrases that describe actions or consequences of actions taken by others towards the analyzed keyword “health”.

The advantage of employing this methodology is that it not only provides insight into the specific health-related motivations that prompted the participants to engage with the project, but also allows for the observation of the contexts in which these motivations are discussed. The capacity to observe the context in which a given motive is discussed allows for the interpretation of the attitudes and intentions held by the participants. Statements belonging to the “associations” category, namely, what a keyword is associated with and what it accompanies, are interpreted differently. This is also true of statements belonging to the “oppositions” category, namely, what the respondents perceive as the opposite of health, and what health is opposed to. Conversely, statements belonging to the category of “actions towards the subject” permit the interpretation of the actions taken by respondents towards the subject, namely, their attitudes towards health.

The analysis also utilized the method of assigning one of the three emotional temperatures [23] to each semantic field of the analyzed keyword “health”: (1) positively charged field; (2) negatively charged field; or (3) neutrally charged field. The classification of positively valued fields was based on the terms in the semantic field that indicated such a character of the utterance, for example, “I like to eat” and “it is nice to eat lettuce”. Conversely, negatively valued fields were classified based on terms such as “I can’t convince myself to eat these things”. Finally, statements that could not be considered positive or negative were classified as neutral. 

In addition to the qualitative approach, the study employed quantitative methods of data analysis. The number of individual semantic fields categorized was counted and presented in descending order of frequency of occurrence. The analysis was conducted using MAXQDA 2022 Analytics Pro software. The research material used for analysis, in the form of interview transcripts, was prepared in Polish, in the language of the interviews.

## 4. Results

### 4.1. Characteristics of Positive-Emotional Networks

#### 4.1.1. Actions towards the Subject

**Figure 1:**
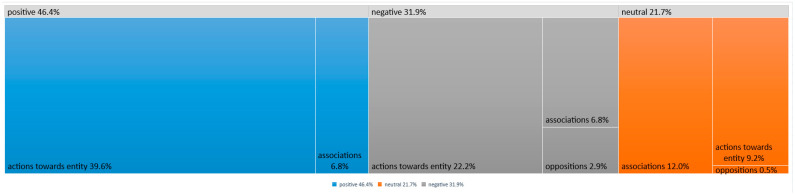
Statements with positive/neutral/negative emotional temperature.

**Figure 2:**
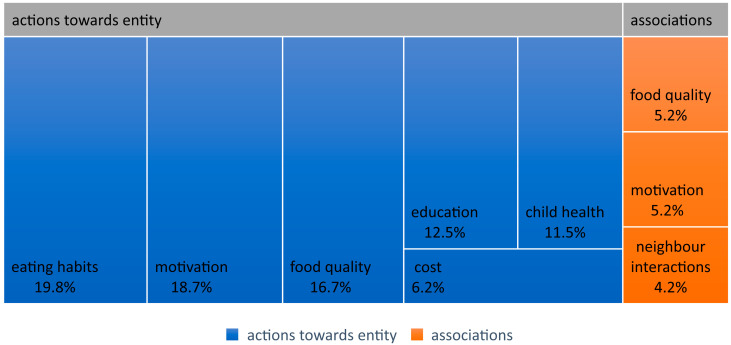
Statements with positive emotional temperature.

Among the most frequently mentioned positive opinions regarding health-related issues were those belonging to the category of “Actions toward the subject”. They constituted 39.6% of all statements (Figure 1). Among them, those concerning dietary habits predominated (19 statements, Figure 2). Participants stated that their decision to modify their diet was usually influenced by aspects related to healthcare through proper nutrition. Access to their own vegetables results in a feeling that the food consumed is clean and healthy. Participants pointed out the need to consume balanced and healthy meals as direct regulators of the overall state of the body or hormone levels. Some participants declared that they take care of the quality of the food they consume, for example, by eating “a healthier variety of pasta” (TG25:249) or maintaining a healthy diet by avoiding supermarkets and shopping at local vendors.

Participants in the interviews also emphasized the impact of their diet on health-related issues associated with appearance. An example could be a statement regarding the practice of drinking celery juice for six months as a “brilliant treatment” (TG30:41) for the skin or the need to “regain part of the wardrobe” (TG37:120). Equally important for this group of participants was the preparation of meals themselves, which is perceived as control over what goes into the proverbial pot, equivalent to perceiving the food consumed as healthy (TG40:193).

Another dominant set of statements concerned motivation to participate in the project (18 statements). Experiment participants indicated that easy access to vegetables would be a reason to change their dietary habits and eat more healthily. Interviewees believed that having access to homegrown vegetables would lead to actual consumption of those vegetables, as there would be no need to buy them, and their freshness and self-grown nature would make them more desirable to eat. Participants also expressed the belief that if such organizational solutions were more popular, more people would be persuaded to adopt healthier eating habits by consuming more vegetables daily.

Another aspect related to motivation to participate in the project that participants declared was the desire to take part in an interesting project. Due to increasing media interest in the topic of growing vegetables and edible plants in urban settings, such as on balconies or terraces, the opportunity to participate in a hydroponic cultivation project served as an incentive to engage in activities related to growing plants, which were perceived as healthy in this respect, “I will just produce healthy food myself” (TG27:161). The attractiveness of the project, in the opinion of the block residents participating in the experiment, lies in recognizing the potential health benefits associated with their “small oasis of nature” (TG37:270).

Due to their frequency, another important health-related aspect was actions related to ensuring good quality food products, mentioned 16 times. Participants were convinced that the vegetables they grow themselves, observing how they grow, are definitely healthy (TG3:149). In the participants’ opinion, these vegetables represent a “cumulation of all mineral nutrients and vitamins” (TG39:186). Another cited method of verifying the quality of the product was using a mobile application supporting healthy shopping choices (TG15:147).

According to the values and beliefs passed down from generation to generation, they defined healthy food as unprocessed (TG4:35). Fruits and vegetables from local suppliers also enjoy high trust. Interview participants talked about practices where they collectively order organic products from such suppliers. Based on the cooperative established by the residents, vegetables and fruits were regularly delivered to them directly from farmers (TG11:315). Examples of actions aimed at obtaining healthy products also included buying flour directly from the producer (TG15:143) or purchasing healthy food at local markets (TG26:459).

Among the participants’ statements, aspects related to learning were also important. This constituted every eighth positive statement. One participant even specified that they are excessively interested in what is inside their food. They regularly read or listen to broadcasts about various nutrients, where they come from, why they are important, what products should be combined, and when they are recommended. They try to incorporate appropriate fats into their diet, avoid excessive carbohydrates, and maintain a balanced diet (TG28:72). Another participant declared that they try to dedicate time to reading about products, especially about health novelties. They have a habit of searching for such products under the influence of suggestions from the Internet or Instagram, for example, while observing dedicated dietary profiles, and then searching for and buying those products in local stores (TG29:55).

One of the participants noticed that the trend of growing edible plants on balconies or terraces is increasing because residents do not want to consume chemically sprayed vegetables. They want access to clean and healthy food. In their opinion, although these people do not have the necessary knowledge about self-cultivation, “they don’t even do it wrong” (TG6:25). For many people, participation in the project is an opportunity not only to acquire knowledge about growing edible plants and gardening skills, but also knowledge about the cultivated plants and how they affect human health (TG30:119).

Educational issues were important regarding participation in the project. Participation in the project may lead participants to read more about healthy food and try various other ways to gain interesting knowledge, not only about growing edible plants but also about the nutrients they contain (TG24:34). Another important action for children’s health undertaken by the interview participants was activities related to caring for children. Among the positive statements, aspects related to children’s health appeared 11 times. These statements emphasize a change in priorities regarding healthy eating habits when a child is born (TG22:358), especially when the child has health problems, for example, allergies (TG15:42). Showing concern for the quality of children’s nutrition is their health, represented by resistance to potential diseases (TG22:358). But also, the health of the child is seen as a response to their food preferences. Parents see good health in children as a result of meeting their expectations by buying the kind of food the child likes and enjoys eating, such as ham without skin and soft bread (TG37:120).

Another significant action for children’s health is their education. Taking care of their health means teaching them how to eat. From the perspective of participating in the project, it is an opportunity to teach children about the growth cycle of plants, and how to take care of plants. As a result, children can learn practically how to produce food without the use of chemicals (TG23:101).

Another positive aspect of perceiving health issues was through the prism of saving money in the household budget (these statements numbered six). In their opinion, consuming healthier food will result in not having to spend money on vegetables (TG27:199). Furthermore, as the participants note, many stores currently have sections with food from organic farming, but they are much more expensive, so growing vegetables themselves will positively affect household expenses (TG30:57). An example cited by one of the participants is the price of watercress, which is considered the healthiest plant. However, it is only available in some stores and at a high price. Participation in the project will thus enable greater access to this particularly appreciated source of nutrients (TG39:233).

#### 4.1.2. Associations

Among the most frequently mentioned opinions regarding health issues, which can be classified as having a positive emotional temperature, were those belonging to the category of “Associations”. They accounted for 14.6% of all positive statements. Among them, those related to statements about factors motivating participation in the experiment predominated (five statements). Among these statements, connections were seen amongst participants’ desire to grow their own edible plants, which equals healthier eating (TG8:205), and the desire of residents to obtain their own vegetables, which will have a positive impact on their health or nutrition (TG22:346), including more diverse vegetables (TG13:279).

Further associations regarding health issues can be made with participants’ statements regarding the quality of food products (five statements). Here, among other things, participants pointed out that it is important for them to have contact with healthy food, i.e., unprocessed food that can be observed growing (TG14:81). It is important for vegetables to be chemical-free and as organic as possible, simply to be as healthy as possible (TG23:101). Also important were aesthetic issues, so that dishes prepared with homegrown vegetables would be well presented (TG37:188). The quality of products was also evaluated based on the association with the form of sale; self-grown will surely be better than packaged (TG40:570).

Health issues also appeared in four statements from residents regarding their neighborly interactions. Topics related to health, healthy eating, and nutrients are often discussed during short encounters in the hallway or in front of the block (TG31:179), or during newly established acquaintanceships (TG15:147). Participation in the experiment is also seen as an opportunity to talk to neighbors about topics such as a healthier lifestyle, reducing chemicals in products, consuming vegetables, and herbs (TG37:138).

Among the statements with a positive emotional temperature, there were none that could be assigned to the category of “Oppositions”.

### 4.2. Characteristics of Neutral-Emotional Networks

#### 4.2.1. Associations

**Figure 3:**
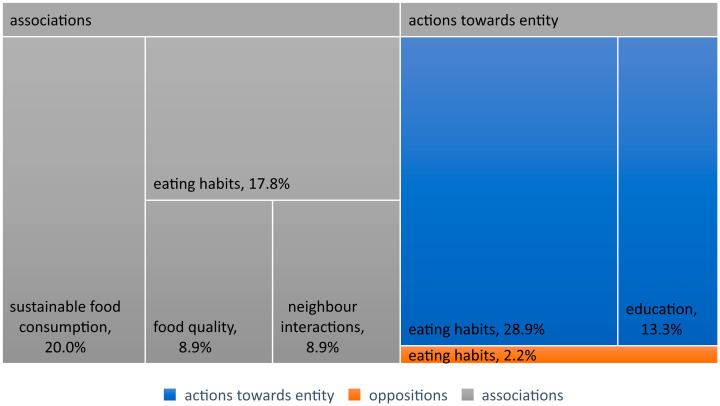
Statements with neutral emotional temperature.

Among the most frequently mentioned opinions regarding health issues, which can be classified as having a neutral emotional temperature, were those belonging to the category of “Associations”. It contained 45 such statements (Figure 3). Interestingly, most issues related to healthy food appeared in the context of responses to the question of what sustainable food consumption is. Participants responded that it is consumption where people eat healthily and consciously, without buying excessive amounts of food that they then throw away (TG14:100). Sustainable food consumption is such that if it is healthy and green, it does not harm (TG40:488). But there were also associations with sustainable consumption, such as healthy eating consisting of “providing the body with what it needs and in the quantities that are needed” (TG38:257).

Next, a significant group (nine statements) consisted of opinions that could be classified into the category of “Eating Habits”. In the participants’ statements, there were fragments indicating that healthy eating is associated with consuming less sugar (TG19:215), more vegetables (TG16:188), longevity (TG22:358), and traditional Polish dishes, as one of the participants believed that good, Polish, healthy cuisine is the best and healthiest (TG37:180).

The next group of statements with a neutral emotional temperature concerned “Neighborly Interactions”. Just like in the case of clearly positive statements, they mainly revolved around health topics discussed during conversations between neighbors during chance encounters. Health issues also arose in relation to the “quality of products”. Here, statements appeared regarding vegetables grown as part of the project, as healthy plants, i.e., those that contain vitamins, are not sprayed with chemicals, so they can be eaten without harm (TG6:94).

#### 4.2.2. Actions towards the Subject

The next most frequently mentioned opinions regarding health issues with a neutral connotation belonged to the category of “Actions toward the subject”. This category contained 19 statements. Among them, the largest group (13 statements) consisted of opinions that could be classified into the “Eating Habits” group. They included participants’ experiences regarding the influence of other people or factors on their eating habits. For some, these included a doctor’s recommendation (TG6:90) or a partner’s suggestion (TG40:512), while for others, they were concern for the state of our planet (TG14:187), general concern for their own health (TG22:358), a specific disease like diabetes, requiring a specific diet (TG40:209), proximity to good health food stores (TG28:148), but also the decision to participate in the project as a stimulus for change (TG16:186).

Another group of statements related to educational activities (six statements). They were mostly concerned with motivation to participate in the project. Within it, participants declared that they mainly want to find out what the proportion of meat, vegetables, and fruits is in their daily diet, what proportion vegetables should occupy in it, and whether they eat healthily as a result. Participants would like to verify their existing knowledge and confirm whether what they think is healthy is indeed healthy (TG5:163). The necessity of caring for plants, and responsibility for them, will motivate them to acquire knowledge about them (TG19:189).

#### 4.2.3. Oppositions

Among the least frequently mentioned opinions regarding health issues, which can be classified as having a neutral emotional temperature, were those belonging to the category of “Oppositions”. There was one statement regarding eating habits. One of the interviewees described times when he ate improperly, and did not follow any diet, as unhealthy times. However, he still retains habits related to eating spicy products such as hot peppers, radishes, horseradish, and garlic. This participant hopes that during the project, his daughter will see him eating such vegetables from his own cultivation and will also want to try them, thus learning about different tastes (TG37:120).

### 4.3. Characteristics of Negative-Emotional Networks

#### 4.3.1. Actions towards the Subject

**Figure 4:**
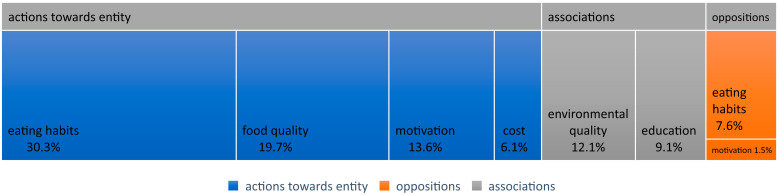
Statements with negative emotional temperature.

Among the most frequently mentioned opinions regarding health issues, which can be classified as having a negative emotional temperature, were those belonging to the category of “Actions towards the subject”. It contained 46 such statements (Figure 4). Among them, the largest group (20 statements) consisted of opinions that could be classified into the “Eating Habits” group. Predominant among them were statements about the lifestyle and eating habits of the interview participants. They declare that even though they try to maintain a healthy diet, from time to time they consume meals that are not healthy (TG16:188), meaning heavy, mainly fried meals, with large amounts of meat, and especially sweets (TG25:248), or their diet is not rich in vegetables (TG18:116). Some respondents expressed the opinion that although they like to eat healthily, they do not like to prepare meals or do not have time for it. They buy unhealthy products to save time (TG22:354), or consume unhealthy meals that are easy and quick to prepare (TG33:151). Participants also question the quality of purchased products. Although they try to read labels, they either do not fully trust the content of the product to match the description on the label, or buy a particular product despite the ingredients not meeting their expectations (TG21:107), often not bringing good food from home and buying something quickly during a break at work (TG36:158).

Among these are statements regarding the difficulties in selecting diets for individuals with health issues. Just as devising a diet for a healthy person usually does not involve significant obstacles, it is challenging for those who are ill to find specialists who specialize in tailoring diets for specific medical conditions (TG9:149). According to participants, taking medications can pose a challenge, as some of them may interact. For example, one participant presented the possibility of adverse reactions when taking blood pressure medication and consuming large amounts of chokeberry juice (TG30:55). Another participant pointed out the difficulty in selecting a diet for hormonal issues (TG32:105).

Another group of statements referencing health issues pertained to the quality of products (13 statements). Participants were critical of the offerings in stores. For instance, participants observed that contemporary jams contain a significant amount of enhancers, while sugar is replaced with substances that are carcinogenic (TG6:105). Deli meats and meats are unhealthy because they contain substantial amounts of chemicals and antibiotics, and are fatty, which adversely affects cholesterol levels, leading participants to abstain from these products (TG11:119). According to participants, vegetables are currently sprayed with chemicals, so buying, for example, tasty tomatoes, is almost impossible (TG37:266). Another example cited a television program whereby a reporter was employed at a bakery and witnessed improper food practices, such as adding chemical agents to baked goods (TG40:207).

Nine statements with a negative emotional temperature appeared regarding the motivation to grow edible plants as part of participating in the experiment. One participant expressed uncertainty about the project’s nature, particularly regarding healthy eating, so they could not determine if the project could change anything in their daily lives. They doubted that merely growing edible green plants as residents would alter their eating habits. According to them, everyone has such strong habits that they are rather difficult to change (TG3:274). Another participant expressed concern about her husband. She assessed that her participation in the project would probably not encourage her husband to join gardening activities because he prefers urban life and would rather spend his free time cycling (TG15:153).

Some statements regarding health appeared in responses regarding motivation to grow plants in general. Participants indicated impatience with plants, and even if they wanted access to healthy food, the plants they grew wilted very quickly, so they preferred to buy them ready-made at the store, such as basil (TG30:49).

Another group of statements with a negative emotional temperature concerned the cost of products. Participants pointed out the very high cost of organic products. Even if they are healthier, they indicated that purchasing them is not worth the extra cost, as their price is often double that of similar non-eco-labeled products (TG26:545). They expressed a similar attitude towards ready-made meals. If they have to pay three times more for a healthy meal than for “junk food”, they prefer to eat fast food (TG28:148). Participants indicated that often it is not a matter of choice, but a necessity, to buy cheaper products (TG36:158).

#### 4.3.2. Associations

Among the frequently mentioned opinions regarding health issues that can be classified as having a negative emotional temperature were those belonging to the category of “Associations”. It contained 14 such statements. Among them, a larger proportion (8 statements) consisted of opinions that could be classified into the environmental quality group. Participants pointed out that modern times are unhealthy because the entire environment is contaminated. For example, they cited the problem of most food products being sold in plastic packaging, resulting in them containing microplastics and heavy metals hazardous to health (TG6:97). Despite attempts to introduce EU regulations regulating chemical content in products, spraying still contaminates groundwater and soil (TG38:98). Participants also expressed opinions such as “I can’t heal the whole world” (TG15:200).

Another group of statements referring to health issues concerned “knowledge”. Dominant among them were opinions about the lack of awareness and knowledge in the current society about the chemicals contained in sold fruits and vegetables (TG25:294). Some participants clearly stated that they see shelves of organic food in stores but are not particularly interested in the subject (TG31:113). Preparing healthier food requires more involvement, searching for recipes, and experimenting, so their dietary monotony is a result of a fast-paced lifestyle, although they declare that it bothers them (TG33:151).

#### 4.3.3. Oppositions

Among the opinions regarding health issues that can be classified as having a negative emotional temperature were also those belonging to the category of “Oppositions”. It contained six such statements. Among them, five statements could be classified into the “Eating Habits” group. Among the participants, those who identified unhealthy eating habits as being associated with a specific period of their lives stood out. It consisted of relying on fast-food dishes and a fast-paced lifestyle (TG37:114). This broader issue was developed in statements that explained the cause of such an unhealthy lifestyle, i.e., in the “Actions towards the subject” category.

One of the statements classified into the “Oppositions” category relates to motivation to participate in the project. One of the respondents clearly emphasized that healthy food is not the reason why they would want to grow it. For this participant, participating in the project is not only fun, but also a form of escape from city life, a glimpse of the possibility of returning to work on the land, a slice of land in the city (TG26:346).

## 5. Discussion

Among the interviewed respondents, the early adopters who expressed a willingness to participate in an innovative experiment aimed at growing their own vegetables and herbs using hydroponic cabins placed in the corridors of their apartment block, three main groups of people can be distinguished. The first group is composed of individuals who are very positively inclined towards health-related issues. They declare a high level of commitment to nutritional matters. Their preferences may result from a general increase in awareness of the benefits of such a lifestyle [28]. On the one hand, they take active steps to prepare and consume balanced and healthy meals in their daily diet. Their efforts are focused not only on preparing meals themselves, but also on sourcing ingredients from smaller local suppliers. Through participation in the project, these participants aspire to enjoy similar access to fresh and healthy vegetables and herbs as users of traditional urban gardens [20]. This access is ensured by independent growth control, which eliminates the harmful heavy metals and pesticides found in commonly available produce [21].

The diffusion of innovations and the formation of environmentally conscious urban communities are processes that are greatly influenced by education, so issues related to the learning of future experimental participants were of interest to the study. Findings indicate that education plays an important role in the lives of individuals in the first group. They emphasized that they dedicate a lot of time to reading, watching programs, listening to broadcasts about nutritional components, optimal food combinations for health, seeking information on balanced and varied diets tailored to their individual needs, as well as health news. This confirms the relationship whereby conscious education has a positive impact on self-care and the environment [29]. Participation in the project has the potential to allow one to deepen their knowledge of food, nutrition, and health topics, as well as acquiring additional related skills that they can implement in their daily practice. Of particular importance to them was the aspect of intergenerational social learning, namely, the opportunity to instill positive habits in their children and peer learning through neighborhood integration for the sake of caring for a shared quality of life and health.

From the perspective of the diffusion of innovations theory, their existing knowledge can be a valuable input for peer learning. Through conversations with others, which are intensified through participation in the intervention, there will be a deeper process of sharing knowledge and experiences [23]. As individuals who are particularly sensitive to health issues and concerned about ensuring a variety of nutrients in their diet, they can have a positive influence on their neighbors’ behavior in this regard. Furthermore, they can encourage not only the consumption of a more balanced diet, but also the cultivation of a variety of plants that are less common or less commonly available in shops, but richer in nutrients. One respondent cited watercress as an example of such a less-common plant that could be cultivated.

The second group consists of individuals who have changed their dietary habits under the influence of other people or factors. For some, it was a suggestion from a doctor or partner, for others, a specific illness necessitating a particular diet, or simply concern for the state of our planet. The third group consists of individuals who, due to their lifestyle, do not attach great importance to health issues. This is most often due to a lack of time, which is also confirmed by other studies [30]. Representatives of this group buy unhealthy products for the sake of convenience, or consume unhealthy meals that are easy and quick to prepare. The intention of these individuals is to increase motivation and opportunities to acquire knowledge about healthy eating through participation in the project.

Representatives of each group were critical of the quality of products available in stores. Available products in large chains contain enhancers, carcinogenic substances, antibiotics, or heavy metals [21]. In their opinion, these products spoil relatively quickly, which increases food wastage. Participation in the project may therefore be a source of healthy food that is unavailable to them on a daily basis. A very important issue with a negative emotional temperature, recurring in the statements of participants, is the high cost of products prepared or grown in ecological conditions. It is the main barrier to consuming these products in their daily diet. For some participants, healthy products are clearly inaccessible due to their price, resulting in them replacing them with unhealthy alternatives, including fast-food dishes. Access to healthy products is a clear factor that influenced the decision to participate in the project.

## 6. Conclusions

The main contribution of this study is in helping us to better understand the health-related motivations, attitudes, intentions, and expectations of participants in an interdisciplinary project aimed at the self-production of food in urban settings. Through a unique educational social experiment, residents of a selected block in Warsaw have the opportunity for the hydroponic cultivation of vegetables and herbs in the corridors of their apartment block. The qualitative study revealed the individual profiles of project participants, individuals with a clearly pro-health lifestyle, individuals for whom health issues are the result of the actions of other people or factors, and busy individuals who declare a lack of time to care for their health. For each of these groups, the opportunity to access healthy products and educational benefits were factors that influenced the decision to participate in the project.

This study indicates that, despite the difficulties in conducting a social experiment, city residents are willing to participate in such innovative activities. It is therefore worthwhile to continue seeking ways to finance unconventional educational interventions utilizing the latest technological solutions. Through them, not only do residents’ access to healthy, nutritious products increase, but it also has the potential to motivate them to lifelong learning, influencing their environmental literacy. The findings of the study indicate that a significant proportion of urban dwellers have limited access to healthy and nutritious fruits and vegetables. This is due to a number of factors, including restrictions related to urbanization and the displacement of local suppliers, as well as lifestyle and the fast pace of urban life. The dissemination of innovative solutions could help to enhance awareness, prompting reflection on healthy habits and strengthening motivation to change eating habits.

However, the study is not without limitations. What on the one hand undoubtedly constitutes a strong point of the project—the uniqueness of the experiment—also represents a research limitation, due to the sample size and the deterministic nature of the study—qualitative interviews, the results of which cannot be generalized. On the other hand, the study aims to understand the profiles of individuals who by their nature constitute a small part of the community—early adopters in the process of technological innovation diffusion; therefore, the size of this group is significantly limited, and quantitative studies would not have a clear justification. Future research could be expanded to include a comparative aspect, comparing different groups of individuals who undertake the testing of similar technological solutions in other geographic locations.

This paper presents the results from the initial phase of the project, prior to the participants being included in the experimental phase. Consequently, the attitudes and intentions of the initial users were expressed prior to the innovation testing period. Further research will be conducted on a number of related issues, including the learning process during the adoption of an innovation, the productivity of urban hydroponic farming, and the sharing of food with neighbors, family, and friends. These findings will be presented in subsequent papers.

The results of the study can serve not only other researchers interested in understanding issues related to healthy eating or implementing technological innovations, but also educators interested in promoting a positive attitude towards urban gardening, as well as food safety, diverse, balanced nutrition, and health.

## Data Availability

The data presented in this study are available on reasonable request from the corresponding author.
